# Thermodynamic characterisation of urban nocturnal cooling

**DOI:** 10.1016/j.heliyon.2017.e00290

**Published:** 2017-04-03

**Authors:** Zhi-Hua Wang, Qi Li

**Affiliations:** a School of Sustainable Engineering and the Built Environment, Arizona State University, USA; b Department of Earth and Environmental Engineering, Columbia University, United States

**Keywords:** Thermodynamics, Environmental science, Energy, Engineering

## Abstract

Nocturnal cooling of urban areas governs the evolution of thermal state and many thermal-driven environmental issues in cities, especially those suffer strong urban heat island (UHI) effect. Advances in the fundamental understanding of the underlying physics of nighttime UHI involve disentangling complex contributing effects and remains an open challenge. In this study, we develop new numerical algorithms to characterize the thermodynamics of urban nocturnal cooling based on solving the energy balance equations for both the landscape surface and the overlying atmosphere. Further, a scaling law is proposed to relate the UHI intensity to a range of governing mechanisms, including the vertical and horizontal transport of heat in the surface layer, the urban-rural breeze, and the possible urban expansion. The accuracy of proposed methods is evaluated against *in-situ* urban measurements collected in cities with different geographic and climatic conditions. It is found that the vertical and horizontal contributors modulate the nocturnal UHI at distinct elevation in the atmospheric boundary layer.

## 1 Introduction

Urban heat island (UHI) study is a central topic in urban climate research (Oke, 1976; Landsberg, 1981). The presence of excessively elevated temperature in city cores, as compared to their rural surroundings, is the main driver of many adverse urban environmental problems, ranging from thermal discomfort and human health (Kovats and Hajat, 2008), to building energy efficiency (Akbari and Konopacki, 2005; Wang et al., 2016), to air quality degradation (Taha, 1997; Sarrat et al., 2006). In addition, field observations as well as model simulations have shown that the UHI effect is often more prominent during nighttime (Chow et al., 2012; Song and Wang 2016a, b). This is especially true for densely built cities with downtown areas full of skyscrapers and narrow and deep street canyons (e.g. New York city, Hong Kong, Tokyo, etc.) or cities in arid and semi-arid cities (e.g. Phoenix) where their urban cores can even be cooler than surrounding rural areas and exhibit the urban “oasis” effect during daytime (Brazel et al., 2000).

The contrast between daytime and nocturnal thermal field patterns in cities has been attributed to multiple players. The plausible contributors include the urban geometry and radiative exchange (Nunez and Oke, 1976; Nakata and de Souza, 2013), enhanced cooling by evapotranspiration in built environment (Miao and Chen, 2014; Yang et al., 2015), and/or landscaping and soil moisture availability (Wang, 2014). In particular, the cooling of the urban land surface is strongly modulated by molecular processes, viz. heat conduction and viscous dissipation around the soil-atmosphere interface, leading to the so-called surface urban heat island (SUHI) phenomenon (Peng et al., 2012), as in contrast to the conventional UHI measured by air temperature anomaly. It remains a challenging task to fully unravel the underlying physics of both UHI and SUHI phenomena for sustainable urban planning and development, e.g. for mitigating thermal stress, improving human thermal comfort, enhancing building energy efficiency, *etc*. (Lazzarini et al., 2015).

Last decades have seen increasing research effort to characterize the underlying physical mechanisms of nocturnal UHI, including numerous *in-situ* measurement campaigns (see e.g. Muller et al., 2013 for a recent review), laboratory experiments and scaling (Uno et al., 1988; Lu et al., 1997a, b; Cenedese and Monti, 2003), remote sensing imagery (Voogt and Oke, 2003), or numerical modelling (Raman and Cermak, 1975; Richiardone and Brusasca, 1989; Chen et al., 2011). The challenge remains open in order to disentangle the energetic mechanisms of nocturnal UHI and SHUI, especially when they are blended in the complex land surface and atmospheric dynamics and their interactions. Specifically, there is a lack of consistent numerical algorithm based on same physical principle (e.g. conservation of thermal energy) in treating both phenomena. Most operational models for UHI study were developed based on one major physical scheme with imbedded (and usually oversimplified) complexity of urban geometry, landscape properties, and flow patterns using empirically determined parameterization (e.g. Oke, 1981; Lee et al., 2012; Nakata and de Souza, 2013). For instance, Oke (1981) treated nocturnal UHI based on a transient cooling model and incorporated the effect of urban geometry in the sky-view factors for estimating the longwave radiative heat transfer.

In this study, we characterise the thermodynamics of the nocturnal cooling of cities using two sets of numerical tools. The first one is the solution of energy balance equation for both urban landscape surface and the overlying atmospheric boundary layer (ABL), each being simplified according to the governing physical processes, e.g. no advection presented at the land surface and molecular processes negligible outside the viscous sublayer. The use of unified principle for energy conservation, therefore, enables analytically-tractable formulation of the dynamic (transient) cooling for both skin (surface) and atmospheric temperatures over built landscapes. The second numerical tool, viz. the mechanistic scaling model, is then used to relate the UHI intensity to the environmental complexity and flow patterns in the nocturnal boundary layer (NBL), based on the UHI characterization by the dynamic model but assuming a quasi-static thermal equilibrium for steady state UHI evolution. In particular, the proposed models in this study will enable us to quantify the impact of urban expansion at the climatological scale.

## 2 Methodology

### 2.1 Surface temperatures and transient SUHI intensity

Consider a generic urban landscape, e.g. a road, a paved surface, a building wall or roof, or a lawn, with exchange of thermal energy at the surface. By surface, we refer to the infinitesimally thin (in theory) solid-air interface plus a thin-layer of near-interface atmosphere that can be delimited, e.g. by the viscous sublayer. The surface energy balance (SEB) is given by Eq. (1) below:

(1) $$R_{n}=H+LE+G$$

where *R_n_* = *S*_net_ + *L*_net_ is the net radiative heat with *S*_net_ and *L*_net_ the net shortwave (solar) and longwave (terrestrial) radiation respectively, and *H*, *LE*, and *G* the sensible, latent and ground heat respectively. Note that the anthropogenic heat emission, though an important energy budget in the built environment (Sailor, 2011), is absorbed into the sensible and latent heat fluxes when urban SEB is considered. This is particular true when ground-based measurements, e.g. eddy-covariance towers, are used in determining SEB, as sensing instruments typically do not distinguish between heat fluxes arising from anthropogenic or non-anthropogenic sources. At the surface, heat transfer is governed by molecular processes, viz. heat diffusion in the solid medium and molecular conduction in the viscous sublayer.

To analytically track the heat conduction process in solid medium, a Green’s function approach can be adopted (Wang et al., 2011; Yang and Wang, 2014), and the subsurface temperature distribution for the urban landscape is given by the Duhamel’s Principle (Carslaw and Jaeger, 1959) as

(2) $$T\left(z,t\right)=T_{i}+\int_{0}^{t}Q_{v}\left(t-\tau \right)dg\left(z,\tau \right)$$

where *z* and *t* are vertical coordinate (positive downwards) and time, *T_i_* is the initial temperature, *Q_v_* is the total vertical heat flux transferred through the solid-air interface, and *g* is the Green’s function (fundamental) solution for heat conduction. The fundamental solution is derived based on the homogeneous surface forcing, prescribed by a heat impulse of unity strength incident at the surface, viz. *Q_v_* = *h* (*t*), where *h* is the Heaviside step function. One series representation of the Green’s function solution is given by (Carslaw and Jaeger, 1959):

(3) $$g\left(z,t\right)=\frac{2\sqrt{\left(\kappa t/\pi \right)}}{\lambda }\exp\left(-\frac{z^{2}}{4\kappa t}\right)-\frac{z}{\lambda }\text{erfc}\left(\frac{z}{2\sqrt{\kappa t}}\right)$$

where *λ* and *κ* are the (molecular) thermal conductivity and diffusivity, respectively, and erfc is the complimentary error function.

In particular, for nocturnal cooling of a generic landscape, surface energy flux is dominated by the releasing of ground heat in the solid medium and the net longwave radiation in the near-surface air. Thus the nocturnal surface energy balance can be approximated by *L*_net_ = *G* = *Q_v_*; both remains roughly time-invariant, especially under calm and clear conditions (Nunez and Oke, 1976). Thus the *skin* temperature *T_s_* can be approximated by combining Eqs. (2)–(3), as (Wang et al., 2011)

(4) $$T_{s}\left(t\right)=T_{i}+2L_{net}\frac{\sqrt{\left(\kappa t/\pi \right)}}{\lambda }$$

Note the time origin *t* = 0 is set at sunset. Eq. (4) is the approximated solutions of surface temperature of a generic landscape during the nocturnal cooling, given that the net radiative cooling flux and the thermal properties of the landscapes are known. For simplicity, we denote $\phi =\frac{2\sqrt{\kappa t/\pi }}{\lambda }$ as the thermal function signalling the contribution of net surface flux to nocturnal cooling of surface temperature. Further denote the intensity of SUHI as the difference between surface temperatures of an urban and a rural landscape, we have the evolution of surface urban heat island as a function of nocturnal cooling as

(5) $$\text{SUHI}\left(t\right)={\left(\text{SUHI}\right)}_{i}+\left(L_{net,\text{urb}}\phi_{\text{urb}}-L_{net,\text{rural}}\phi_{\text{rural}}\right)$$

where (SUHI)_i_ is the initial SUHI intensity at the onset of nocturnal cooling. It is clear that the nocturnal SUHI is governed by the co-evolution of vertical fluxes arising from urban-rural landscapes and their temporal thermal function *ϕ*, respectively.

### 2.2 Air temperatures, urban-rural breeze, and transient UHI intensity

In the atmospheric surface layer overlying a land surface, the general energy balance equation is given by the conservation of thermal energy (Stull, 1988), which is reproduced in Eq. (6) below:

(6) $$\rho_{a}c_{p}\frac{DT_{a}}{Dt}=\lambda \nabla^{2}T_{a}-\nabla \left(R_{n}+LE+{\underset{˜}{Q}}_{t}\right)$$

where $D/Dt=\partial /\partial t+\underset{˜}{U}\cdot \nabla$ is the Laplacian (total) derivative with $\underset{˜}{U}$ the mean wind vector, *ρ*_a_ and *c_p_* the density and specific heat of air, and ${\underset{˜}{Q}}_{t}=\rho_{a}c_{p}\left(\bar{{\underset{˜}{u}}^{\prime }{T^{\prime }}_{a}}\right)$ the eddy-diffusive (turbulent) heat flux vector. In particular, outside the viscous sub-layer, the molecular heat transfer process becomes insignificant as compared to the turbulent transfer. In addition, as the nocturnal cooling episode of an urban area is considered, evaporation (and latent heat transfer) is largely suppressed by the stable atmospheric stratification, and the divergence of radiative heat in the surface layer is small. For simplicity, we further choose our coordinate system such that the longitudinal direction *x* is aligned with the mean horizontal wind direction, such that mean wind at lateral and vertical direction vanishes, i.e. *V* = *W* = 0. With the aforementioned assumptions, the atmospheric energy balance equation in the surface-layer overlying an urban area can be simplified as (Lee et al., 2012),

(7) $$\frac{\partial T_{a,\text{urb}}}{\partial t}+U\frac{\partial T_{a,\text{urb}}}{\partial x}=q^{\prime \prime }$$

where $q^{\prime }=\nabla \left(\bar{w^{\prime }{T^{\prime }}_{a}}\right)$ is the divergence of eddy-diffusive heat.

**Fig. 1:**
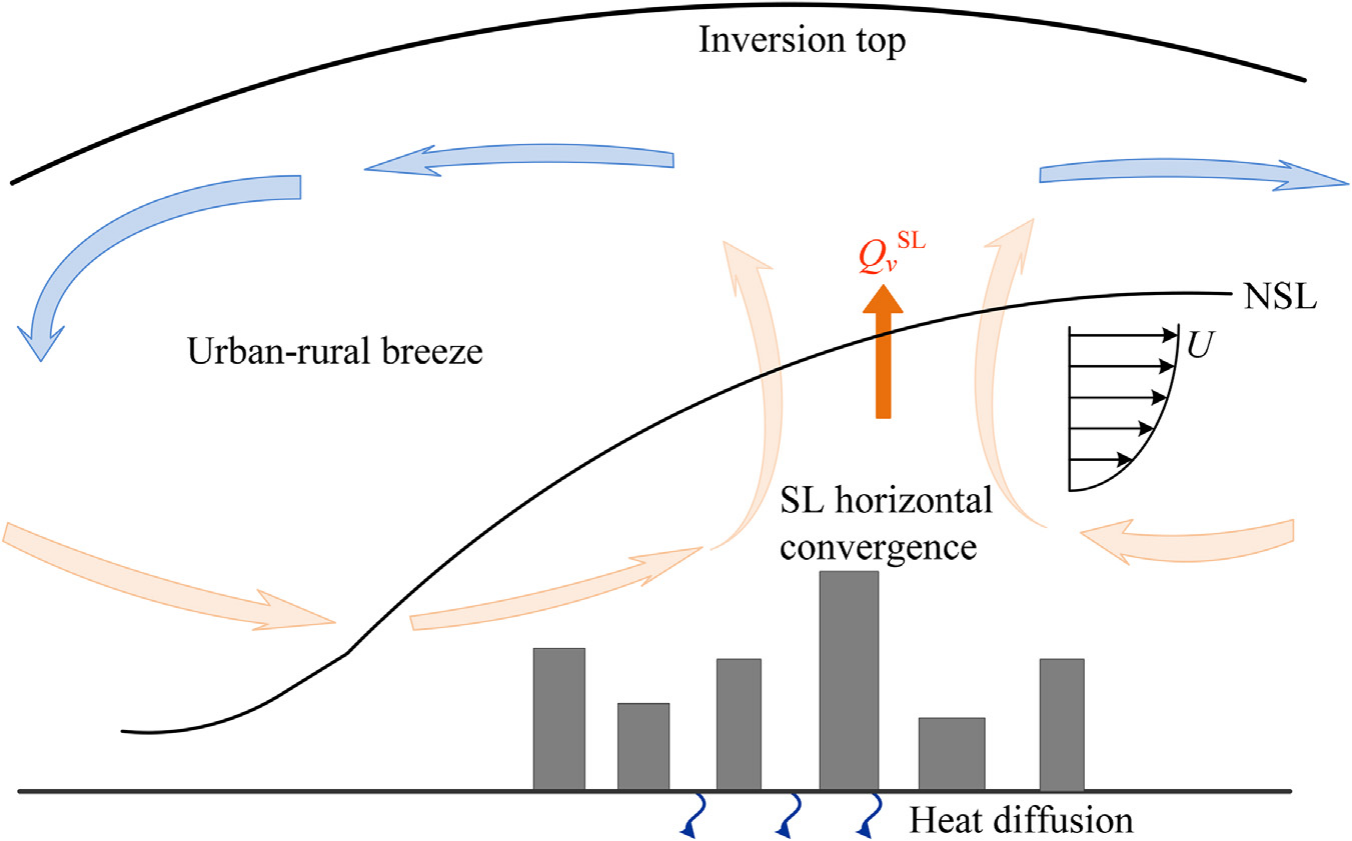
Schematics of governing mechanisms for nocturnal cooling of an urban area and contribution to urban heat island (NSL: nocturnal surface layer, $Q_{v}^{\text{SL}}$: vertical heat flux arising from the surface layer).

The advection term in Eq. (7) is strongly related to the air flow pattern in the atmospheric surface layer, known as the urban-rural breeze (Fig. 1), primarily due to the excessive temperature in urban areas caused by UHI. Thus Eq. (7) can be further approximated as

(8) $$\frac{\partial T_{a,\text{urb}}}{\partial t}\approx -U\frac{T_{a,\text{urb}}-T_{a,\text{rural}}}{L_{c}}+q^{\prime \prime }$$

where *L_c_* is the characteristic length of a city. For most cities of irregular morphology, *L_c_* can be approximated as $L_{c}=\sqrt{A/4\pi }$ where *A* is the surface area of the city that can be measured or estimated by remote-sensing techniques, e.g. the Landsat Thermal Mapper (Landsat TM).

The simplified energy balance in Eq. (8) admits two particular solutions, corresponding to limiting cases. First, in the limiting case of no horizontal advection, viz. *U* = 0, we have

(9) $$\Delta T_{a}=T_{a,\text{urb}}-T_{a,\text{rural}}=\Delta T_{a,i}+q^{\prime \prime }t$$

assuming that the divergence of turbulent heat is independent of the mean temperature evolution in the light of spectrum gap. It is clear that the urban-rural breeze leads to a convergence of heat flux in the atmospheric surface layer, i.e. $q^{\prime \prime }>0$ in general, which in turn enhances the nocturnal urban-rural temperature difference, viz. the nocturnal UHI intensity. In the other the limiting case $q^{\prime \prime }=0$, we obtain a second particular solution as

(10) $$\Delta T_{a}=\Delta T_{a,i}\exp\left(-\frac{U}{L_{c}}t\right)$$

Denote that UHI = Δ*T_a_* = *T_a_*_,urb_ − *T_a_*_,rural_ is the UHI intensity (analogous to the definition of SUHI, c.f. Eq. (5)). To derive a general solution of Eq. (8), here we follow the method in Lee et al. (2012), and combine the two particular solutions in Eqs. (9)–(10) by retaining the functional form of exponential decay for UHI intensity, as

(11) $$\text{UHI}={\left(\text{UHI}\right)}_{i}\exp\left(-\psi \frac{U}{L_{c}}t\right)$$

where *ψ* is the lumped factor accounting for the impact of $q^{\prime \prime }$: In the limiting case $q^{\prime \prime }$ = 0, *ψ* = 1, and in general *ψ* < 1 to reflect the heat convergence resulted from urban-rural breeze in the surface layer. Typical values of *ψ* = 0.7∼ 0.9 are used for nocturnal cooling by urban-rural breeze, following Lee et al. (2012). Moreover, an increase in mean wind field has the apparent effect in enhancing the transport of heat away from a city and reducing the thermal stress in the urban core. On the other hand, urban expansion gives rise to the increase in city size characterized by *L_c_*, which in turn retards the heat dissipation in urban atmosphere, leading to higher UHI intensity.

In addition, it is noteworthy that the scaling of SUHI in Eq. (5) and the scaling of UHI in Eq. (11) are analogous in that they are both formulated as the synergistic effect of the initial intensity combined with thermal-temporal functions. These thermal-temporal functions are governed by landscape thermal properties and the vertical flux in the case of SUHI scaling and by the city size and the horizontal flow pattern in the case of UHI scaling, and both decay (approximately exponentially) during nocturnal cooling as time elapses.

### 2.3 Scaling of steady-state UHI

The above-developed models for SUHI and UHI intensities are based on the energy balance equations and are transient (time-varying) in nature. As comparison, in the history of UHI study, an alternative group of methods were developed based on “scaling”, viz. relating the UHI intensity with its causal variables (energetic and aerodynamic contributors) (Lu et al., 1997a, b; Cenedese and Monti, 2003). The scaling method develops functional relationship between these variables (usually in dimensionless groups) largely based on observational dataset acquired in laboratory setting, under steady state of temperature anomaly evolution. This steady state UHI in laboratory measurements therefore implicitly assumes a thermal equilibrium between the development of urban temperature field and the vertical and horizontal fluxes (together with the wind velocity and stability conditions). A sample estimate of UHI intensity using mechanistic scaling law is given by (Lu et al., 1997a, b; Lee et al., 2012) as

(12) $${\left(\text{UHI}\right)}_{\text{steady}}={\left(\frac{2Q_{h}^{SL}D_{c}\partial \theta /\partial z}{\rho_{a}c_{p}U}\right)}^{1/2}$$

where $Q_{h}^{SL}$ is the horizontal heat flux convergent into the urban core in the surface layer, *D_c_* the characteristic UHI diameter, and ∂θ/∂z is the vertical gradient of potential temperature (measured above the urban core. Comparison of Eq. (12) to Eq. (11) indicates that besides the apparent difference in time-dependences of UHI evolution, all causal effects of UHI development, e.g. the urban-rural breeze, the temperature (or flux) gradient, the mean wind field, the urban size, and the thermal properties are presented in both formulation, though in different functional forms.

## 3 Results and discussion

### 3.1 Characterization of SUHI intensity

**Fig. 2:**
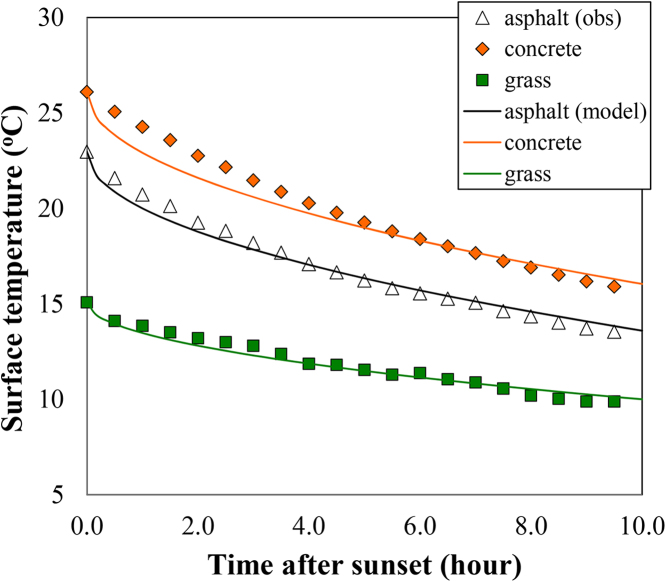
Comparison of observed and model predicted average nocturnal skin temperatures of generic landscape types including asphalt and concrete pavements and urban lawn (short grass), at Princeton during 04–09 May 2010.

We first test evaluate the analytical scheme for nocturnal cooling of surface temperature and the associated SUHI intensity in Eq. (5) against observational data, based on Eq. (4) for surface temperature calculation. The measurement dataset was recorded during a 2-year field campaign at Princeton NJ, using a network of wireless weather stations (Sensorscope®). Measurement of skin temperatures over 3 types of landscape, viz. a concrete road, an asphalt pavement, and a urban lawn of short grass, during the sample period of 6 days, 04–09 May 2010, was used for model validation in the figure (see Muller et al., 2013 and Wang et al., 2013 for more details of field instrumentation and data collection). Thermal properties of the three landscapes are specified as *κ*_con_ = 6.7 × 10^−7^ m^2^ s^−1^, *λ*_con_ = 1.2 W m^−1^ K^−1^, *κ*_asp_ = 1.0 × 10^−6^ m^2^ s^−1^, *λ*_asp_ = 1.6 W m^−1^ K^−1^, *κ*_veg_ = 4.0 × 10^−7^ m^2^ s^−1^, and *λ*_veg_ = 0.8 W m^−1^ K^−1^. The net longwave radiation is approximated as time-invariant with *L*_net_ = −70 W m^−2^. The results of comparison of observed and model-predicted nocturnal cooling of the 3 distinct landscape types, averaged over the 6-day measurement period, are shown in Fig. 2. It is clear that despite its simplicity, the analytical model predicts the evolution of surface temperatures of all 3 different (and typical) urban landscapes during nocturnal cooling with good accuracy, with the root-mean square errors (RMSEs) well below 1 °C.

**Fig. 3:**
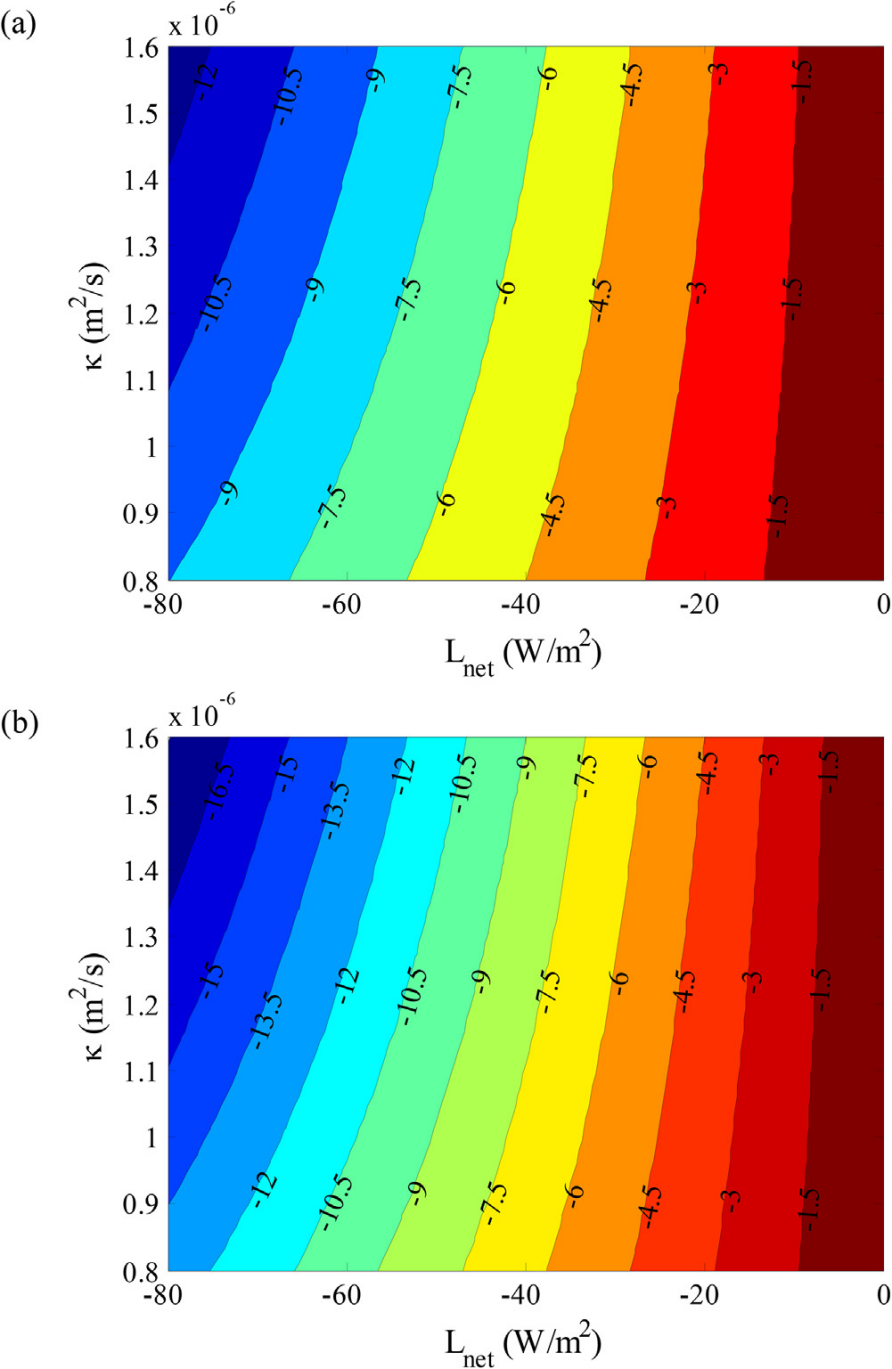
Contour plot of transient SUHI intensity as a function of net vertical flux and thermal properties of the urban landscape at (a) 5 h, and (b) 10 h after sunset respectively.

Next we proceed to quantify the relative contribution of urban thermal properties and the net vertical flux at the urban surface to the transient SUHI intensity, i.e. the nocturnal cooling of urban area corresponding to the second term of Eq. (5) (with the thermal properties specified above), assuming the nocturnal cooling of its rural counterpart happens independently. The results are plotted in Fig. 3 for a range of net longwave radiation and thermal diffusivity over urban area, as these are the control parameters in the simplified analytical model in Eq. (5) for SUHI scaling. The upwelling *L*_net_ ranges from 0 to −80 W m^−2^ and the thermal diffusivity *κ*_urb_ ranges from 0.8 × 10^−6^ m^2^ s^−1^ to 1.6 × 10^−6^ m^2^ s^−1^; both corresponding to ranges of physical variation of the parameters. The estimated nocturnal cooling of urban surface increases with the magnitude of net upwelling longwave radiation and decreases with the molecular diffusivity. The impact of vertical flux is much more significant than the thermal property of the landscape, given that at a fixed *L*_net_, the variability of surface cooling due to change of diffusivity (with maximum cooling of 3.7 °C and 5.3 °C for *L*_net_ = −80 W m^−2^ at 5 h and 10 h after sunset, respectively) is much less than the cooling variability induced by changing *L*_net_ (c.f. maximum cooling of 12.8 °C and 18.1 °C at 5 h and 10 h after sunset, respectively at *κ*_urb_ = 1.6 × 10^−6^ m^2^ s^−1^). This observation, together with the fact that a well-established built environment admits only small degree of variability of landscape properties (e.g. via infrastructure retrofit), apparently suggests that the intensity of nocturnal SUHI is predominated by meteorological conditions, unless large scale city redevelopment is envisioned.

### 3.2 Scaling of UHI intensity and the impact of urban expansion

**Fig. 4:**
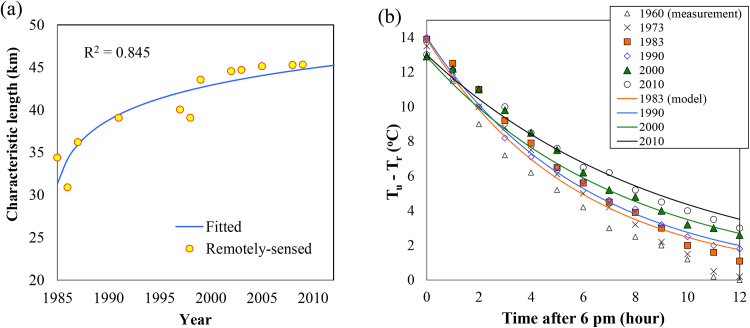
Urban expansion of Phoenix metropolitan area and its impact on nocturnal cooling: (a) historical evolution of characteristic length of the city, and (b) comparison of model predictions and measurements of nighttime cooling.

The transient model of UHI intensity, descried in Section 2.2, is compared with field observation at the Phoenix metropolitan. Fig. 4(a) shows the evolution of characteristic urban length, *L_c_*, estimated based on the remotely-sensed imagery by ASTER from 2002 to 2010 and Landsat TM images prior to 2000 (Lee et al., 2012). The scattered observational dataset is then fitted with a logarithmic function, with a goodness-of-fit value of 0.845. The fitted *L_c_* curve thus allows us to bridge the gap in observational data, as well as to predict plausible future urban growth scenario in the study area. The estimated *L_c_* values were then used in Eq. (11) to predict the nocturnal cooling of urban air as a function of time. The results of comparison of the modelled and observed nighttime UHI for years 1983, 1990, 2000, and 2010 are shown in Fig. 4(b), as annual averages. The model is capable of predicting the evolution of nocturnal UHI (as temperature difference between air temperatures at the urban core of Phoenix and the surrounding rural environment) with reasonable good accuracy. It is shown that the continuous expansion of the Phoenix metropolitan area in last few decades has led to retarded urban cooling during nighttime and a more prominent residual UHI at dawn.

Furthermore, to evaluate the relative contribution of horizontal and vertical fluxes to UHI intensity, we use the steady-state scaling law in Eq. (12) and re-group the causal variables such that

(13) $${\left(\text{UHI}\right)}_{\text{steady}}=\sqrt{2\Delta T_{v}\Delta T_{h}}$$

where $\Delta T_{h}=\frac{Q_{h}^{SL}}{\rho_{a}c_{p}U}\frac{D_{c}}{L_{m}}$ and $\Delta T_{v}=L_{m}\frac{\partial \theta }{\partial z}$ are the horizontal and vertical temperature anomaly scales, corresponding to heat fluxes induced by horizontal flow convergence and the vertical temperature gradient in the ABL overlying the urban environment, respectively; and *L_m_* is the vertical mixing length (Sun, 2011). More specifically, as our primary interest in this study is in nocturnal cooling of urban areas at meteorological scale, we will focus on the two major contributors in the above UHI scaling, viz. horizontal *U_m_* and vertical ∂θ/∂z, both are strong functions of the elevation. Further, we resort to the Monin-Obukhov similarity theory (MOST) to parameterize these variables as function of *z* (Stull, 1988), as

(14) $$\kappa \frac{z}{u_{*}}\frac{dU}{dz}=\phi_{m}\left(\zeta \right)$$

where *κ* = 0.4 is the von Karman’s constant, u_*_ is the surface friction velocity, *ζ* = *z*/*L* the dimensionless stability length scale with *L* the Obukhov length, and *φ_m_* the stability structural function. For simplicity, in subsequent analysis, we assume the nocturnal ABL is nearly neutrally stratified ($\zeta \approx 0,\phi_{m}\approx 1$ in Eq. (14)), such that the vertical distribution of the mean wind speed follows the well-known log-law profile,

(15) $$U=\frac{u_{*}}{\kappa }\ln\left(\frac{z-d}{z_{0,\text{urb}}}\right)$$

where *d* is the zero-plane displacement and *z*_0,urb_ is the roughness length by treating built structures in the urban environment as canyons of bluff elements. These geometric properties can be approximated as *d* = *H*/2, and *z*_0,urb_ = 2*H*/3 in Eq. (15) where *H* is the average building height (Masson, 2000). Similarly, using MOST, the vertical temperature gradient in the neutral urban surface layer is given by Eq. (16) as

(16) $$\frac{\partial \theta }{\partial z}=\frac{\theta_{*}}{\kappa \left(z-d\right)}=-\frac{Q_{v}^{SL}}{\rho_{a}c_{p}\kappa u_{*}\left(z-d\right)}$$

where $\theta_{*}=-Q_{v}^{SL}/\rho_{a}c_{p}u_{*}$ is the surface layer temperature scale, and $Q_{v}^{SL}$ is the vertical heat flux in the surface layer that is negative (upwelling). The two temperature scales in Eq. (13) are obtained in Eqs. (17)–(18) as,

(17) $$\Delta T_{h}=\frac{Q_{h}^{SL}D_{c}}{\rho_{a}c_{p}L_{m}}\frac{\kappa }{u_{*}}\frac{1}{\ln\left[\left(z-d\right)/z_{0,\text{urb}}\right]}$$

and

(18) $$\Delta T_{v}=-L_{m}\frac{Q_{v}^{SL}}{\rho_{a}c_{p}}\frac{1}{\kappa u_{*}\left(z-d\right)}$$

and the ratio of the two is given by Eq. (19),

(19) $$\beta =\frac{\Delta T_{v}}{\Delta T_{h}}=\frac{L_{m}^{2}\ln\left[\left(z-d\right)/z_{0\text{,urb}}\right]}{D_{c}\kappa^{2}\left(z-d\right)}\left(-\frac{Q_{v}^{SL}}{Q_{h}^{SL}}\right)$$

**Fig. 5:**
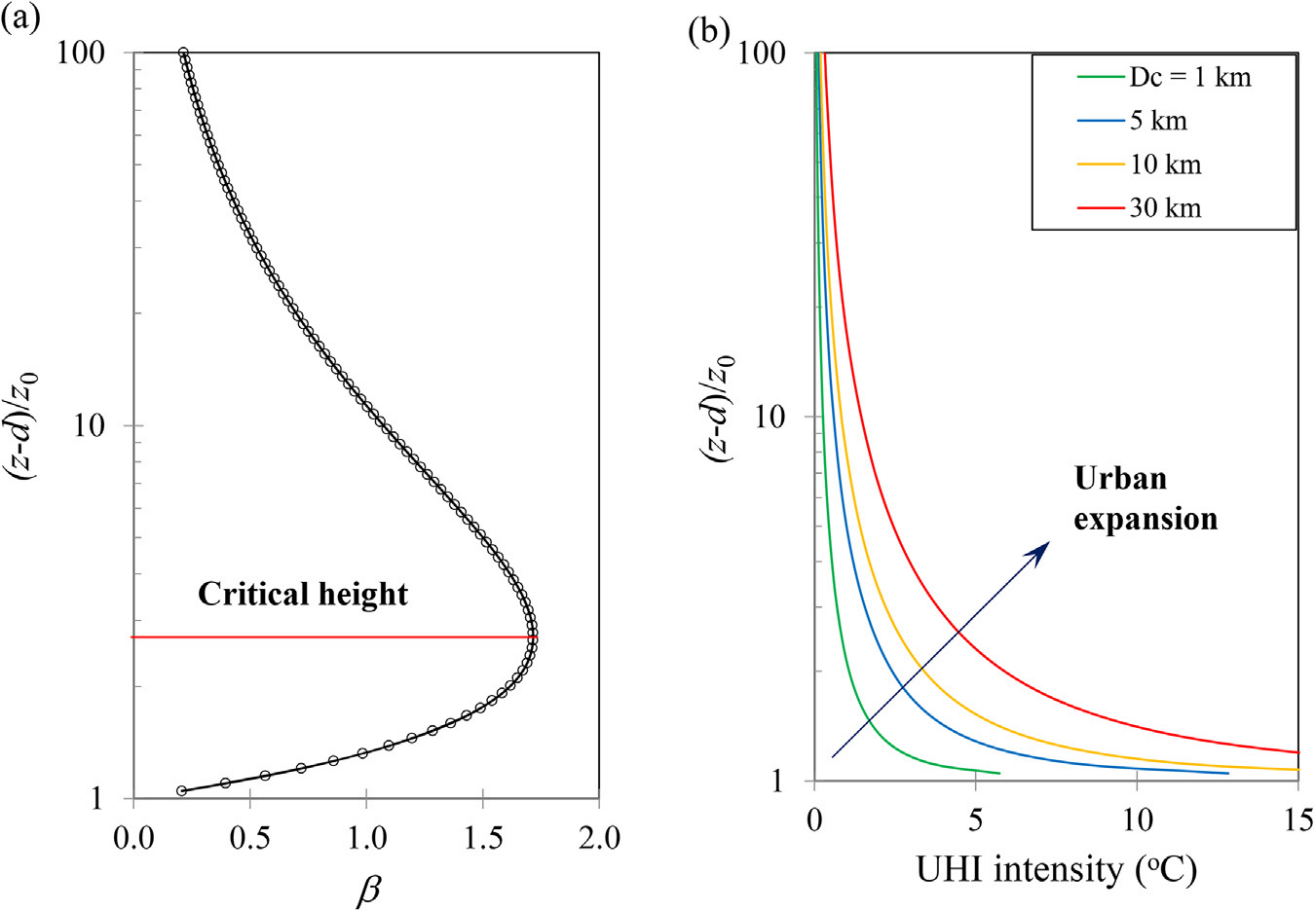
Results of steady-state UHI scaling: (a) the vertical-to-horizontal temperature scale ratio as a function of elevation, and (b) nocturnal UHI intensity as a function of characteristic city size.

Furthermore, to illustrate the UHI scaling, we conducted a case study here using the following set of parameters: *L_m_* = 50 m, *D_c_* = 1000 m, u_*_ = 0.4 m s^−1^, *ρ_a_* = 1.2 kg m^−3^, *c_p_* = 1005 J kg^−1^ K^−1^, *z*_0,urb_ = 6.7 m, *d* = 5 m, $Q_{v}^{SL}$ = −50 W m^−2^, and $Q_{h}^{SL}$= 25 W m^−2^. The choice of the parameters is not completely arbitrary, but represents typical values within physical ranges as observed in nocturnal surface layers (NSL) over built terrains and properties of the atmosphere. The dependence of the vertical-to-horizontal temperature scale ratio *β*, as a function of elevation, is plotted in Fig. 5(a). It is apparent that the ratio increases with the elevation first till it reaches a critical height, above which *β* decreases with elevation. The determination of the critical elevation is straightforward by taking ${\left(d\beta /dz\right)|}_{z=z_{c}}=0$, so we have

(20) $$z_{c}=d+ez_{0,\text{urb}}\text{or}\frac{z_{c}-d}{z_{0,\text{urb}}}=e=2.718$$

The critical height in Eq. (20) turns out to be a constant ratio of the urban roughness. The trend of variation of the *β* ratio suggest that, in general, near the surface, the UHI intensity is more likely to be dominated by the temperature gradient and vertical heat flux (e.g. radiative cooling of the surface), while above the critical elevation, the nocturnal UHI is more strongly regulated by the horizontal advection (e.g. urban-rural breeze).

In addition to the meteorological scale analysis, it is also of interest to see how, in the long run, prospective urban expansion, either due to well-planned city development (like the Phoenix metropolitan in the US) or due to less well-planned rapid urbanization in many developing countries, can affect the UHI phenomenon. Using the simple scaling law in Eq. (13), the predicted *nocturnal* UHI intensity is shown in Fig. 5(b), where all parameters are the same as in Fig. 5(a) except the varying characteristic UHI diameter *D_c_*. It is clear that as the city expands (represented by increasing *D_c_*), the nocturnal UHI is expected to exacerbate, particularly in the near surface atmospheric layer, where unfortunately, is also the space where vast majority of human activities occur. The total UHI intensity, as a synergistic effect of vertical divergence and horizontal urban-rural breeze, decreases with elevation in general, but is able to “penetrate” to higher elevation in larger cities (Hu and Brunsell, 2015; Song and Wang, 2015).

## 4 Conclusions

In this study, we developed numerical algorithms for charactering thermodynamics of nocturnal UHI and SUHI, in both dynamic (based on transient energy balance) and steady state (based on scaling laws) settings. In particular, the proposed scale model enables us to quantify the relative contribution of vertical and horizontal heat flux arising from the urban NSL. It is found that the more localized vertical radiative cooling dominates the UHI intensity up to a critical height $\left(z_{c}-d\right)/z_{0,\text{urb}}=e$, above which the larger scale urban-rural breeze and horizontal advection take the control. In addition, the impact of urban expansion on UHI at climatological scale can also be characterized using the proposed model, indicating a strong warming potential for SUHI and near-surface UHI intensities, whereas its influence decays rapidly at higher elevation (e.g. in the nocturnal residual layer). The results of this study provide useful guidance to sustainable urban planning for decision makers, landscape managers, practitioners as well as individual property owners, especially with design and implementation of UHI/SUHI mitigation strategies. This is particularly useful for cities located in arid/semiarid environment, where nocturnal cooling is of practical importance in enhancing human thermal comfort for outdoor activities during nighttime.

Nevertheless, we iterate here that the derivation of the proposed model, especially for the analytically-based dynamic algorithms, was based on a number of simplifications. For example, urban landscapes are essentially treated as slabs in nature, while the complexity in urban morphology is blended in the simple representation of roughness length (c.f. Oke, 1981 where canyon aspect ratios were used to represent urban geometry). In addition, the proposed model does not admit any strong anisotropy, either in landscape properties (spatial coverage, material properties, *etc*.) or in the dynamics of flow (urban-rural breeze, advection, *etc*.). Thus we expect that due care needs to be exercised for extending the proposed model and the current results to unusual scenarios, e.g. fractal growth of very irregular cities or presence of strong nocturnal jets in the ABL. Potential improvement of the proposed model can be made with more accurate determination of local landscape, thermal, and geomorphological properties, as well as site-specific design based on climatology and socioeconomic conditions at the locality.

Furthermore, we used neutral ABL for deriving the scaling law of UHI intensity in this study. As the urban NBL can be subject to any atmospheric stability conditions, in particular, it tends to be unstable due to horizontal convergence of flux and enhanced vertical transport, it leaves the possibility of future work wide open in investigating the impact of different stability conditions and its contribution to the UHI intensity. Though this can be carried with a more generalized scaling law as the one in this study, for more accurate representation of actual urban-land atmosphere interactions, we recommend that the use of more sophisticated numerical modeling techniques, such as large eddy simulations, will possibly provide more insight into the problems of interest.

## Declarations

### Author contribution statement

Zhihua Wang: Conceived and designed the experiments; Performed the experiments; Analyzed and interpreted the data; Contributed reagents, materials, analysis tools or data; Wrote the paper.

Qi Li: Contributed reagents, materials, analysis tools or data; Wrote the paper.

### Funding statement

This work is supported by the National Science Foundation (NSF) under grant CBET-1435881 and CBET-1444758, and Army Research Laboratory (ARL) under grant W911NF-15-1-0003 and W911NG-16-1-0045.

### Competing interest statement

The authors declare no conflict of interest.

### Additional information

No additional information is available for this paper.
